# Risk factors for discontinuation of intravenous patient-controlled analgesia after general surgery: a retrospective cohort study

**DOI:** 10.1038/s41598-023-45033-2

**Published:** 2023-10-26

**Authors:** Saeyeon Kim, In-Ae Song, Boram Lee, Tak Kyu Oh

**Affiliations:** 1 Department of Anesthesiology and Pain Medicine, Seoul National University Bundang Hospital, Gumi-ro 173 Beon-gil, Bundang-gu, 13620 Seongnam, South Korea; 2 Interdepartment of Critical Care Medicine, Seoul National University Bundang Hospital, Gumi-ro 173 Beon-gil, Bundang-gu, 13620 Seongnam, South Korea; 3 Department of Anesthesiology and Pain Medicine, College of Medicine, Seoul National University, 103 Daehak-ro, Jongno-gu, 03080 Seoul, South Korea; 4 Department of Surgery, Seoul National University Bundang Hospital, Gumi-ro 173 Beon-gil, Bundang-gu, 13620 Seongnam, South Korea

**Keywords:** Risk factors, Outcomes research

## Abstract

Identifying patients at risk for developing side effects secondary to intravenous patient-controlled analgesia (IV PCA) and making the necessary adjustments in pain management are crucial. We investigated the risk factors of discontinuing IV PCA due to side effects following general surgery; adult patients who received IV PCA after general surgery (2020–2022) were included. Data on postoperative pain intensity, PCA pain relief, side effects, continuity of PCA use, and PCA pump settings were collected from the records of the acute pain management team. The primary outcome was identifying the risk factors associated with PCA discontinuation due to side effects. Of the 8745 patients included, 94.95% used opioid-containing PCA, and 5.05% used non-steroidal anti-inflammatory drug (NSAID)-only PCA; 600 patients discontinued PCA due to side effects. Female sex (adjusted odds ratio [aOR] 3.31, 95% confidence interval [CI] 2.74–4.01), hepato-pancreatic-biliary surgery (aOR 1.43, 95% CI 1.06–1.94) and background infusion of PCA (aOR 1.42, 95% CI 1.04, 1.94) were associated with an increased likelihood of PCA discontinuation. Preoperative opioid use (aOR 0.49, 95% CI 0.28–0.85) was linked with a decreased likelihood of PCA discontinuation. These findings highlight the importance of individualized pain management, considering patient characteristics and surgical procedures.

## Introduction

Adequate control of acute postoperative pain is crucial for optimal recovery, prevention of chronic pain, and reduction of postoperative morbidity^1^. Medical professionals carefully balance the amount of analgesics administered to avoid excessive amounts of opioids, which can result in side effects such as opioid-induced hyperalgesia^1^, nausea, vomiting, hypotension, respiratory depression, postoperative ileus^2^, bradycardia^3^, pruritus^4^, sedation, confusion, and urinary retention^5^. To benefit patients with the smallest effective amounts of opioids, a multimodal approach to analgesia using various routes and different analgesics has gained much attention for enhanced recovery after surgery^2^. However, due to its simplicity, intravenous patient-controlled analgesia (IV PCA) remains essential for inpatient pain management.

IV PCA allows postoperative patients to receive analgesics easily and immediately by pressing a button^6^. The IV PCA infusion pump was programmed with a background infusion rate, bolus dose, and interval between doses to titrate the amount of analgesics that would effectively alleviate pain while minimizing side effects^5^. Despite attempts to account for individual differences in pharmacodynamics and pharmacokinetics, some patients discontinue PCA owing to intolerable side effects. Previous studies have identified several risk factors for developing side effects associated with opioid-containing PCA, which lead to PCA discontinuation. Determining these factors, such as older age^7^, comorbidities^8^, and opioid-naïveté^9^, is essential for managing acute pain after general surgery.

Since studies identifying the risk factors for discontinuing IV PCA—including the use of non-opioids and opioids—are needed, our study aimed to investigate such factors in patients who received IV PCA after general surgery.

## Methods

### Ethical considerations

We followed the Strengthening the Reporting of Observational Studies in Epidemiology guidelines for conducting this retrospective cohort study based on population data^10^.

This study was approved by the Institutional Review Board (IRB) of Seoul National University Bundang Hospital (SNUBH) as a retrospective cohort study conducted at a single tertiary academic hospital (IRB approval number: B-2304-821-002).

The IRB of SNUBH waived the requirement to obtain informed consent from the patients because of the retrospective cohort design, which analyzed the health records of patients who had already completed their treatment.

### Data source and study population

This study used electronic health records from the BESTCare^11^ system and ICD-10 codes to classify comorbidities.

Inclusion criteria comprised adult patients (≥ 20 years) admitted to our hospital between January 1, 2020, and December 31, 2022, who received IV PCA after general surgery. Multiple surgeries for each patient performed on the same date were considered as one.

The following subsets of patients were excluded: those receiving anesthesia other than general or spinal anesthesia, those discharged within 2 days after surgery, and those transferred to the intensive care unit from the operating room. Also excluded were patients lacking the necessary medical information, those not discharged by the end of the study period, and those who died in the hospital.

### Study endpoints (IV PCA discontinuation due to side effects)

In 2020, SNUBH established an acute pain management team consisting of an attending anesthesiologist and a registered nurse from the Department of Anesthesiology and Pain Medicine. This team conducts daily rounds to assess patients receiving PCA to evaluate the adequacy of pain relief, the presence of side effects, and the continuity of PCA use. The team also monitored the daily number of analgesics administered, the number of refills, interventions done to control side effects, and patient satisfaction with the overall PCA use. Moreover, the acute pain management team checked patients who stopped using IV PCA specifically due to side effects such as nausea/vomiting, dizziness, pruritus, sleeping tendency, and respiratory distress, among others. In general, IV PCA was administered for approximately 2 to 4 days after general surgery, and the acute pain management team performed daily checks for PCA discontinuation due to side effects during this period. Using this information, the primary endpoint of this study was identification of the risk factors associated with PCA discontinuation owing to side effects after general surgery.

### Study parameters

Initial nursing records provided patients’ medical history and regular preoperative use of non-steroidal anti-inflammatory drugs (NSAIDs), paracetamol, or opioids. We gathered data on patient demographics, anesthetic methods, surgical details, postoperative length of hospital stay, and in-hospital mortality status from the same records. We also determined whether the surgery was laparoscopic and classified it into one of eight categories: stomach, colon, hepatopancreatic-biliary (HPB), breast, small bowel, transplantation, or vascular surgery.

Information on the American Society of Anesthesiologists (ASA) physical status and comorbidities such as hypertension, diabetes, chronic kidney disease, cardiac disease (coronary artery disease, arrhythmias, valvular heart disease, and heart failure), cerebrovascular disease, and liver disease (cirrhosis, hepatitis, fatty liver, and liver cancer) were obtained from premedication records. These premedication records were registered using interviews with patients or guardians at hospital admissions. Further, data on the PCA setting device, the drug used, and the duration of PCA use (refill and discontinuation) were collected from the PCA assessment records by the acute pain management team. Registered nurses in SNUBH assessed postoperative pain scores using the Numeric Rating Scale at least 4 times daily; however, the data were not collected for this study because it was unclear whether the pain scores were recorded before or after administration of the PCA bolus or other analgesics.

### Statistical analysis

The patients’ baseline demographics were reported as means with standard deviations for continuous variables and as numbers with percentages for categorical variables. The clinicopathological characteristics of the PCA continuation and discontinuation groups were compared using t-tests for continuous variables and chi-square tests for categorical variables. Binary logistic regression, both univariable and multivariable, was performed to analyze PCA discontinuation after general surgery. All covariates were included in the multivariable model for adjustment. The results are presented as adjusted odds ratios (aOR) with 95% confidence intervals (CI). Statistical analyses were performed using IBM SPSS Statistics for Windows (version 27.0, IBM Corp., Armonk, NY, USA) with a *P*-value < 0.05 considered statistically significant.

## Results

### Study population

**Figure 1:**
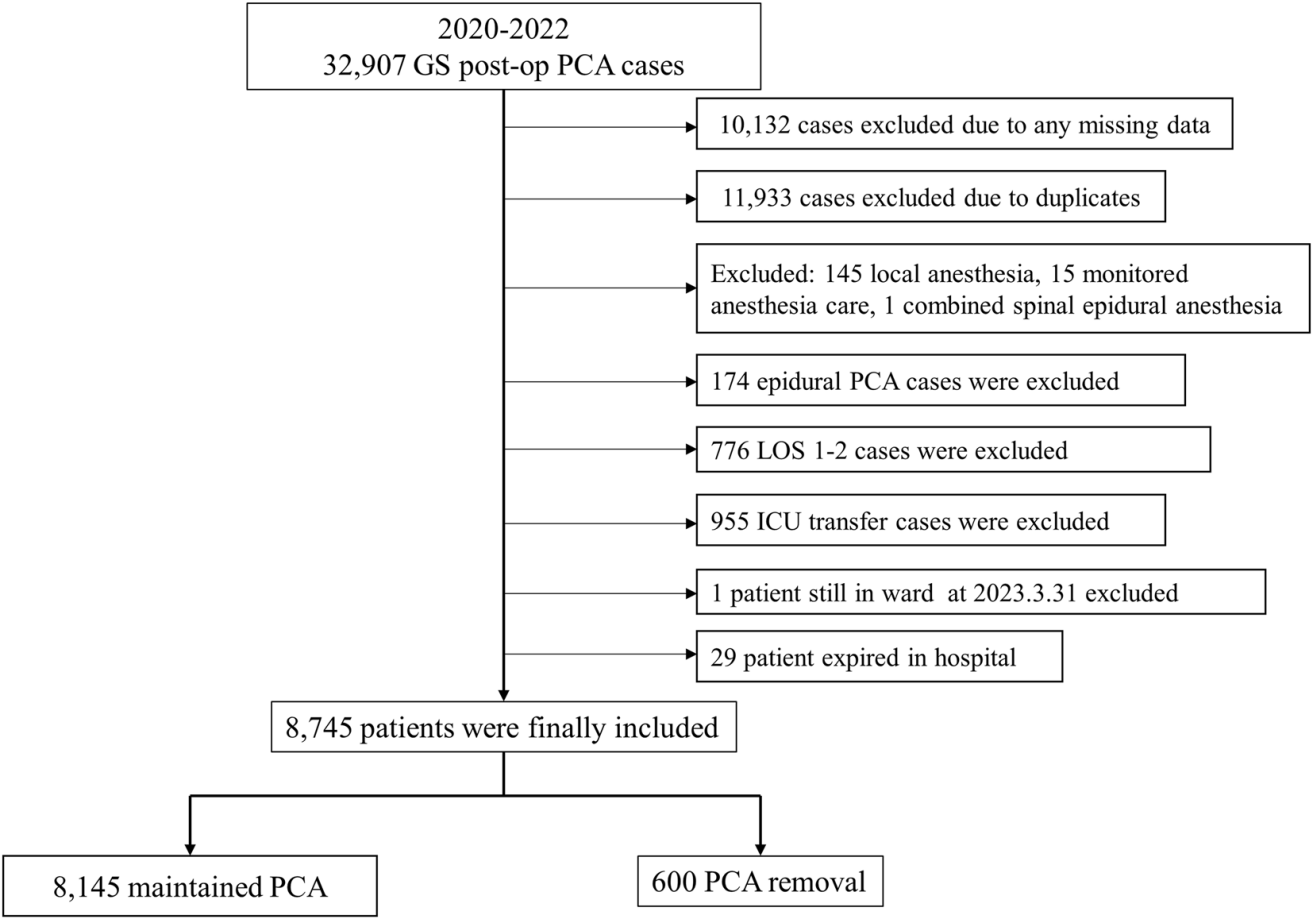
A flowchart of the patient selection process. *GS* general surgery, *PCA* patient-controlled analgesia, *LOS* length of hospital stays, *ICU* intensive care unit.

Between January 1, 2020, and December 31, 2022, 32,907 patients who received a PCA device after general surgery were initially included in the study. A total of 10,132 cases were excluded because they lacked the medical information necessary for the study; 11,933 cases were excluded due to duplicates. Also, 145 local anesthesia cases, 15 monitored anesthesia care cases, and one combined spinal epidural anesthesia case were excluded because we only included patients who received either general or spinal anesthesia. Furthermore, 174 cases of epidural PCA were excluded because we were only interested in patients who received IV PCA. Lastly, the following were also excluded: 776 patients who were admitted for < 3 days after surgery, 955 patients who were transferred to the intensive care unit postoperatively, 29 patients who died in the hospital, and 1 patient who was still admitted at the time of the study. Thus, 8745 patients were included in the final analysis. A flowchart of the patient selection process is shown in Fig. 1 and Table 1 presents the clinical and demographic characteristics of the patients.

**Table 1:** Baseline information of patients.

Variable	Mean (SD) or N (%)
Age, year (mean, SD)	60.65 (14.28)
Female sex—no (%)	4301/8745 (49.18)
LOS after surgery, day	7.28 (9.50)
Medical history—no./total no. (%)	
DM	767/8745 (8.78)
HTN	1313/8745 (15.04)
CKD	218/8745 (2.49)
Heart disease	275/8745 (3.14)
Brain disease	239/8745 (2.73)
Liver disease	678/8745 (7.75)
Anesthesia	
General anesthesia	8716/8745 (99.67)
Spinal anesthesia	29/8745 (0.33)
ASA physical status classification	
1	1539/8745 (17.60)
2	5579/8745 (63.80)
≥ 3	1627/8745 (18.60)
Laparoscopy	5822/8745 (66.58)
Type of surgery	
Stomach	1894/8745 (21.66)
Colon	2391/8745 (27.34)
Hepato-pancreatic-biliary	1244/8745 (14.23)
Breast	1002/8745 (11.46)
Small bowel	357/8745 (4.08)
Transplantation	155/8745 (1.77)
Vascular	62/8745 (0.71)
Others	1640/8745 (18.75)
PCA type	
Opioid included	8303/8745 (94.95)
NSAID only	442/8745 (5.05)
Background infusion	4382/8745 (50.11)
Preoperative drug usage	
NSAID	448/8745 (5.12)
Paracetamol	588/8745 (6.72)
Opioid	640/8745 (7.32)

*LOS* length of hospital stays, *DM* diabetes mellitus, *HTN* hypertension, *CKD* chronic kidney disease, *ASA* American Society of Anesthesiologists, *PCA* patient-controlled analgesia, *NSAID* non-steroidal anti-inflammatory drug.

The patients who received IV PCA after general surgery at SNUBH had a mean age of 60.65 years (standard deviation, SD = 14.28), and their mean length of hospital stay after surgery was 7.28 days (SD = 9.50). Most (99.67%) patients received general anesthesia for surgery, and 66.58% underwent laparoscopic procedures.

The PCA drugs were divided into two groups: those containing opioids (94.95%) and those containing only NSAIDs (5.05%). Preoperative use of NSAIDs, paracetamol, and opioids was recorded to assess their impact on patient outcomes. Of the 8745 patients, 600 (6.86%) discontinued IV PCA due to its side effects. Data were collected on various aspects of PCA, including the device and drug used, PCA settings, effectiveness, discontinuation time, and refills. The PCA device, drug, and settings were determined using a standardized regimen established by the anesthesiologists (Table S1). Factors including the patient’s weight, age, type of surgery, usage of laparoscopy, preoperative laboratory findings, and attending anesthesiologist were also taken into consideration.

### PCA discontinuation

Table 2 presents the differences between the patients who continued using PCA and those who discontinued it. Both groups had a similar health status, as shown by age, comorbidities (except liver disease), and ASA physical status classification. The PCA discontinuation group comprised mostly female patients (71.17% vs. 47.56% in the PCA discontinuation vs. PCA continuation group, respectively; *P* < 0.05). Further, 5400 patients in the PCA continuation group (5400/8145, 66.30%) and 422 patients in the PCA discontinuation group (422/600, 70.33%) underwent laparoscopic surgery (*P* = 0.043). The percentage of patients with opioid-included PCA was higher in the PCA discontinuation group than in the PCA continuation group (96.67% vs. 94.82%, respectively, *P* = 0.046). The percentage of patients with regular preoperative usage of opioids was higher in the PCA continuation group than that in the control group (7.54% vs. 4.33%, *P* = 0.004).

**Table 2:** Comparison of characteristics between the PCA groups.

Variable	PCA continuation (%), N = 8145	PCA discontinuation (%), N = 600	*P* value
Age (SD)	60.68 (14.28)	60.35 (14.20)	0.589
Sex, female	3874 (47.56)	427 (71.17)	< 0.001
LOS after surgery (SD)	7.36 (9.76)	6.21 (4.55)	< 0.001
Medical history			
DM	719 (8.83)	49 (8.17)	0.581
HTN	1231 (15.11)	84 (14.00)	0.461
CKD	207 (2.54)	11 (1.83)	0.283
Heart disease	253 (3.11)	22 (3.67)	0.448
Brain disease	217 (2.66)	22 (3.67)	0.146
Liver disease	613 (7.53)	65 (10.83)	0.003
Anesthesia			0.143
General anesthesia	8116 (99.64)	600 (100.00)	
Spinal anesthesia	29 (0.36)	0 (0.00)	
ASA physical status classification			0.935
1	1434 (17.61)	105 (17.50)	
2	5199 (63.83)	380 (63.33)	
≥ 3	1512 (18.56)	115 (19.17)	
Laparoscopy	5400 (66.30)	422 (70.33)	0.043
Type of surgery			0.016
Stomach	1765 (21.67)	129 (21.50)	
Colon	2246 (27.58)	145 (24.17)	
Hepato-pancreatic-biliary	1137 (13.96)	107 (17.83)	
Breast	951 (11.68)	51 (8.50)	
Small bowel	325 (3.99)	32 (5.33)	
Transplantation	145 (1.78)	10 (1.67)	
Vascular	59 (0.72)	3 (0.50)	
Others	1517 (18.62)	123 (20.50)	
PCA type			0.046
Opioid included	7723 (94.82)	580 (96.67)	
NSAID only	422 (5.18)	20 (3.33)	
Background infusion	4093 (50.25)	289 (48.17)	0.002
Preoperative drug usage			
NSAID	420 (5.16)	28 (4.67)	0.599
Paracetamol	558 (6.85)	30 (5.00)	0.081
Opioid	614 (7.54)	26 (4.33)	0.004

*PCA* patient controlled analgesia, *SD* standard deviation, *LOS* length of hospital stays, *DM* diabetes mellitus, *HTN* hypertension, *CKD* chronic kidney disease, *ASA* American Society of Anesthesiologists, *PCA* patient-controlled analgesia, *NSAID* non-steroidal anti-inflammatory drug.

### Logistic regression

Table 3 shows the results of univariable logistic regression analyses for PCA discontinuation after general surgery. In the adjusted multivariable model in Table 4, where all variables were controlled, being female remained a significant predictor of PCA discontinuation (aOR 3.31, 95% CI 2.74, 4.01; *P* < 0.001). In addition, patients who received background infusion via IV PCA were more likely to discontinue PCA due to side effects than those without background infusion setting (aOR 1.42, 95% CI 1.04, 1.94, *P* = 0.026). Compared to stomach surgery, surgeries related to the HPB (aOR 1.43, 95% CI 1.06, 1.94; *P* = 0.021) and small bowel (aOR 1.68, 95% CI 1.09, 2.60; *P* = 0.019) were more likely to result in PCA discontinuation. On the other hand, breast surgery (aOR 0.57, 95% CI 0.37, 0.86; *P* = 0.007) and preoperative opioid use were linked with a lower probability of PCA discontinuation (aOR 0.49, 95% CI 0.28, 0.85; *P* = 0.012).

**Table 3:** Univariable logistic regression analyses for discontinuation of PCA after general surgery.

Variable	OR (95% CI)	*P*-value
Age, year	1.00 (0.99, 1.00)	0.589
Female sex (vs male sex)	2.72 (2.27, 3.26)	< 0.001
LOS after surgery, day	0.95 (0.93, 0.98)	< 0.001
Medical history		
DM	0.92 (0.68, 1.24)	0.581
HTN	0.92 (0.72, 1.16)	0.462
CKD	0.72 (0.39, 1.32)	0.285
Heart disease	1.19 (0.76, 1.85)	0.448
Brain disease	1.39 (0.89, 2.17)	0.148
Liver disease		
Anesthesia		
General anesthesia	1	
Spinal anesthesia	0.00 (0.00)	0.998
ASA physical status classification		0.935
1	1	
2	1.00 (0.80, 1.25)	0.998
≥ 3	1.04 (0.79, 1.37)	0.786
Laparoscopy (vs laparotomy)	1.21 (1.01, 1.44)	0.043
Type of surgery		0.018
Stomach	1	
Colon	0.88 (0.69, 1.13)	0.321
Hepato-pancreatic-biliary	1.29 (0.99, 1.68)	0.063
Breast	0.73 (0.53, 1.02)	0.069
Small bowel	1.35 (0.90, 2.02)	0.149
Transplantation	0.94 (0.49, 1.84)	0.864
Vascular	0.70 (0.22, 2.25)	0.545
Others	1.11 (0.86, 1.43)	0.427
PCA type		
Opioid included	1	
NSAID only	0.63 (0.40, 1.00)	0.048
Background infusion (vs no background infusion)	1.38 (1.02, 1.85)	0.035
Preoperative drug usage		
NSAID	0.90 (0.61, 1.33)	0.600
Paracetamol	0.72 (0.49, 1.04)	0.082
Opioid	0.56 (0.37, 0.83)	0.004

*LOS* length of hospital stays, *DM* diabetes mellitus, *HTN* hypertension, *CKD* chronic kidney disease, *ASA* American Society of Anesthesiologists, *PCA* patient-controlled analgesia, *NSAID* non-steroidal anti-inflammatory drug.

**Table 4:** Multivariable logistic regression analyses for discontinuation of PCA after general surgery.

Variable	aOR (95% CI)	*P*-value
Age, year	1.00 (0.99, 1.01)	0.989
Female sex (vs male sex)	3.31 (2.74, 4.01)	< 0.001
LOS after surgery, day	0.96 (0.94, 0.99)	0.001
Medical history		
DM	1.01 (0.71, 1.43)	0.975
HTN	0.86 (0.65, 1.15)	0.303
CKD	0.64 (0.25, 1.65)	0.354
Heart disease	1.52 (0.94, 2.46)	0.089
Brain disease	1.55 (0.96, 2.51)	0.074
Liver disease	1.35 (1.00, 1.81)	0.051
Anesthesia		
General anesthesia	1	
Spinal anesthesia	0.00 (0.00)	0.998
ASA physical status classification		0.565
1	1	
2	1.10 (0.88, 1.40)	0.433
≥ 3	1.18 (0.87, 1.62)	0.288
Laparoscopy (vs laparotomy)	1.12 (0.88, 1.41)	0.364
Type of surgery		< 0.001
Stomach	1	
Colon	0.96 (0.74, 1.25)	0.764
Hepato-pancreatic-biliary	1.43 (1.06, 1.94)	0.021
Breast	0.57 (0.37, 0.86)	0.007
Small bowel	1.68 (1.09, 2.60)	0.019
Transplantation	1.82 (0.63, 5.21)	0.267
Vascular	1.38 (0.40, 4.68)	0.610
Others	1.23 (0.94, 1.60)	0.135
PCA type		
Opioid included	1	
NSAID only	0.63 (0.39, 1.03)	0.066
Background infusion (vs no background infusion)	1.42 (1.04, 1.94)	0.026
Preoperative drug usage		
NSAID	0.87 (0.58, 1.31)	0.508
Paracetamol	1.16 (0.69, 1.94)	0.590
Opioid	0.49 (0.28, 0.85)	0.012

*LOS* length of hospital stays, *DM* diabetes mellitus, *HTN* hypertension, *CKD* chronic kidney disease, *ASA* American Society of Anesthesiologists, *PCA* patient-controlled analgesia, *NSAID* non-steroidal anti-inflammatory drug.

## Discussion

This study illustrates the risk factors of discontinuing IV PCA after general surgery. Female sex, PCA with background infusion, and surgery related to HPB and small bowel were factors associated with discontinuing PCA. In contrast, breast surgery and regular preoperative usage of opioids were associated with the continuation of PCA. By identifying patients with these risk factors, medical staff can effectively prevent side effects while providing sufficient analgesia.

In this study, female sex was the most significant risk factor for PCA discontinuation owing to adverse effects. Females have been identified as more prone to postoperative nausea and vomiting^12^ and more likely to experience poor postoperative pain management^12^. These differences may be due to psychosocial factors, such as greater willingness to report pain^13^, or biological factors, such as variations in pain perception^14^. Regardless of the underlying mechanism, females, on average, require 11% more morphine than males for adequate postoperative pain relief^15^. This increased need for analgesics is attributed to frequent PCA bolus doses, which result in a higher incidence of side effects in female patients.

IV PCA is necessary in many hospitals because many hospitals and health systems do not have an acute pain service and may not be capable of delivering epidural or catheter infusion-based analgesia. Moreover, IV PCA provides a level of safety based on lockout intervals for time and dose, reduces nursing burden, and improves patient satisfaction as they don’t need to wait for analgesia. Therefore, it is important to identify high-risk populations for IV PCA discontinuation, such as female patients, because medical staff can promptly address side effects with prophylactic or regular administration of antiemetics or dexamethasone^16^ to prevent the discontinuation of IV PCA. Additionally, alternative analgesic strategies such as PCA composed of ketamine^17^ or nefopam^18^, and nerve blocks suitable for surgery (transversus abdominis plane block, ilioinguinal nerve block, iliohypogastric nerve block, etc.) should be considered. These tailored analgesic plans can alleviate pain and lower the risk of PCA discontinuation due to side effects.

Compared to stomach surgery, HPB surgery was more associated with discontinuation of PCA due to side effects in this study. Patients who underwent major HPB surgery and were admitted for more than 3 days after surgery mostly had generally high complication rates^19^. Further, as HPB surgeries are performed with unavoidable laparotomy, it results in worse postoperative pain^20^. Since pain from this surgery category is severe, many ongoing studies recommend multimodal approaches combining various means such as epidural analgesia^21^, intrathecal analgesia^22^, continuous infusion through a wound catheter^23^, and transversus abdominis plane infiltration^19^. Patients undergoing major HPB surgery may require frequent administration of rescue analgesia, increasing the total amount of analgesics given and aggravating the degree of side effects.

Regular preoperative opioid use is associated with a reduced risk of PCA discontinuation. Despite higher postoperative opioid consumption, opioid-tolerant patients demonstrated fewer adverse effects, except for sedation^24^. In particular, opioid-tolerant patients utilizing PCA report fewer side effects such as nausea, vomiting, and pruritus than opioid-naïve controls^25^. Thus, patients with a history of regular preoperative opioid use may be more likely to experience fewer adverse effects and continue PCA.

A PCA regimen with background infusion was associated with the tendency to discontinue PCA. Previous studies have shown that continuous fentanyl background infusion regimens tend to result in higher total infused fentanyl doses and more frequent side effects, such as dizziness and pruritus^26^. Despite these potential drawbacks, IV PCA offers advantages over other methods of delivering analgesics, such as epidural infusion or PCA.

In the past, without a PCA device, standard IV infusion pumps were used to deliver the drugs epidurally. However, the cessation rate of epidural fentanyl/bupivacaine infusion was about 58%, mostly due to catheter dislodgment, catheter insertion site inflammation, and inadequate analgesia^27^. The rate of epidural PCA discontinuation was 16.22%, mostly due to nausea and dizziness^28^. In our study, about 7.36% of patients discontinued IV PCA due to side effects. A newly developed PCA device with a programmable mode is embedded to address these concerns. This device can adjust the background infusion rate depending on how frequently patients demand bolus doses. It has also been shown to offer improved postoperative analgesia, reduced side effects, and lower the total fentanyl dose infused compared to the conventional PCA regimen^26^. This new program can be applied to patients with the risk factors to minimize side effects while providing effective postoperative analgesia.

This study has several limitations. First, our hospital has a preset PCA regimen determined based on age, weight, surgery type, and the attending anesthesiologist of the day. However, the total amount and type of analgesics were not precisely tailored to each patient, which may have acted as confounding factors. Second, although we identified patients prescribed opioids, NSAIDs, or paracetamol, we could not assess their drug compliance because of the retrospective nature of the study. Third, although we excluded patients who underwent epidural or intrathecal PCA, we could not ascertain which patients received local infiltration of analgesics in the operating room. Fourth, we could not obtain information regarding the amount of rescue analgesics administered besides PCA, which might have influenced the observed and reported side effects. Finally, detailed quantitative data about the adverse effects of IV PCA could not be documented because the accurate frequency of each adverse effect associated with IV PCA discontinuation was not recorded.

In conclusion, effective control of acute postoperative pain is essential for optimal medical outcomes and patient satisfaction. However, concerns about opioid dependence and abuse are widespread, particularly considering the ongoing opioid crisis. Thus, our study aimed to identify and address the risk factors for opioid use and IV PCA at the individual level. A precision medicine approach that identifies high-risk individuals and evaluates targeted and individualized strategies is required to address this issue.

### Supplementary Information

Supplementary Table S1.

## Data Availability

The data that support the findings of this study are available from the corresponding author, TKO, upon reasonable request.
