# Supplementary material for: Risk factors for discontinuation of intravenous patient-controlled analgesia after general surgery: a retrospective cohort study
Source: Sci Rep. 2023 Oct 26;13:18318. doi: 10.1038/s41598-023-45033-2 (PMC10603031; doi:10.1038/s41598-023-45033-2)
Supplement: Supplementary file 1 — Supplementary Table S1. [file 41598_2023_45033_MOESM1_ESM.docx]

Table S1. PCA Regimen for General Surgery

1. Regimen

Pf. A, B

| Age | Weight | Laparotomy & Laparoscopy/Robot | Setting |
| --- | --- | --- | --- |
| <70 | ≥60kg | Fentanyl 18 mcg/ml | Pf. A : (0-1-10)  Pf. B : (1-1-15)  Pf. B stomach surgery : (0-1-10) |
|  | 45~59kg | Fentanyl 15 mcg/ml |  |
|  | <45kg | Fentanyl 12 mcg/ml |  |
| ≥70 | ≥60kg | Fentanyl 15 mcg/ml |  |
|  | 45~59kg | Fentanyl 12 mcg/ml |  |
|  | <45kg | Fentanyl 9 mcg/ml |  |

Pf. C, D, E, F, G, H, I, J

| Age | Weight | Laparotomy | Laparoscopy/Robot | Setting |
| --- | --- | --- | --- | --- |
| <70 | ≥60kg | Fentanyl 18 mcg/ml | Fentanyl 15 mcg/ml | (1-1-15) |
|  | 45~59kg | Fentanyl 15 mcg/ml | Fentanyl 12 mcg/ml |  |
|  | <45kg | Fentanyl 12 mcg/ml | Fentanyl 9 mcg/ml |  |
| ≥70 | ≥60kg | Fentanyl 15 mcg/ml | Fentanyl 12 mcg/ml |  |
|  | 45~59kg | Fentanyl 12 mcg/ml | Fentanyl 9 mcg/ml |  |
|  | <45kg | Fentanyl 9 mcg/ml | Fentanyl 7 mcg/ml |  |

Etc.

| Operation | Regimen | Setting | Device |
| --- | --- | --- | --- |
| Thyroid surgery with robot | Fentanyl 12 mcg/ml | (0-2-15) | ACE320 |
| Breast surgery | *Pf. F :  Ketorolac 3.6 mg/ml  *Patients with kidney disease: ketorolac cannot be used | (1-1-15) | *Ketorolac PCA : accufuser |
| Anal fistulectomy & Hemorrhoidectomy |  |  |  |
| Sleeve gastrectomy |  |  |  |

1. Adjustments

|  | Adjustments |
| --- | --- |
| Pf. A | Patients undergoing liver surgery & AST/ALT ≥120 : 80% of original dose |
| Pf. F, G | 1. ESRD patients : 80% of original dose   (Patients without diagnosis of ESRD but GFR≤40 : check with anesthesiologists)   1. AST/ALT≥200 : check with anesthesiologists |
| Pf. C, D, G, J | Patients diagnosed with CKD or ESRD : 80% of original dose |
| Pf. B, F, H | GFR<60 or OT/PT≥120 : 80% of original dose |
| Pf. E, H | Abnormalities of GFR or BUN/Cr : check with anesthesiologists  GFR≥60: Ketorolac PCA can be applied |

The values are presented as the concentration of fentanyl (bolus volume [ml] – basal rate [ml/hr] – lockout time [min]).

AST aspartate aminotransferase; ALT aspartate transaminase; ESRD end stage renal disease; CKD chronic kidney disease; GFR glomerular filtration rate; BUN blood urea nitrogen levels
